# Mechanistic DFT studies – helicate-type complexes with different alcylic spacers

**DOI:** 10.1186/1758-2946-4-S1-P9

**Published:** 2012-05-01

**Authors:** Verena Gossen, Gerhard Raabe, Markus Albrecht

**Affiliations:** 1 Institute of Organic Chemistry, RWTH Aachen University, Landoltweg 1, 52074 Aachen, Germany

## 

**Figure 1:**
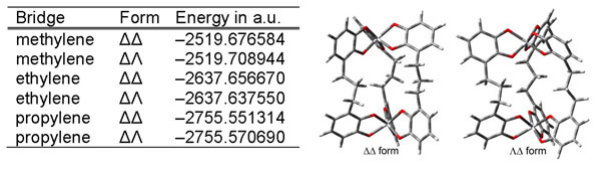
Geometrically optimized complexes with Ti(IV); left: energies of the complexes with different alcylic spacers; right: ΔΔ and ΛΔ form of the complex with a propylene spacer.

Metal-controlled self-assembly of complexes is of high interest in the field of Supramolecular Chemistry [1,2]. In the current study, we synthesized binuclear complexes with different spacers and study the influence of chain length on their relative energy. The considered complexes prefer the zigzag conformation. Thus a bridge with an odd number of methylene units forms a meso-Helicate (ΔΛ or ΛΔ) and one with an even number leads to a Helicate (ΔΔ or ΛΛ) (figure 1) [3,4].

Comparison of the calculated transition energies for the non-dissociative interconversions of the diastereomeres with experimental results provides inside into the isomerization process. Moreover, insertion of different cations (templates) into the cavities of the binuclear complexes and corresponding calculations allow prediction of their influence on the isomerization.

Enlargement of the studied system results in binuclear complexes with imino-bridged ligands. The obtained computational results provide a possible explanation for the experimentally observed high diastereoselectivity.

As the DFT functionals like B3LYP do not describe long-range interactions properly, we chose the coulomb-attenuating method CAM-B3LYP [5] which corrects the exchange interaction at long range. The complexes with Ti(IV) in their helical or meso form have been geometrically optimized at the CAM-B3LYP level of theory with the TZVP basis set and MDF10 as ECP for Ti(IV) as implemented in the program package Gaussian09 [6].
